# Different Analytical Approaches in Assessing Antibacterial Activity and the Purity of Commercial Lysozyme Preparations for Dairy Application

**DOI:** 10.3390/molecules18056008

**Published:** 2013-05-21

**Authors:** Milena Brasca, Stefano Morandi, Tiziana Silvetti, Veronica Rosi, Stefano Cattaneo, Luisa Pellegrino

**Affiliations:** 1 Institute of Sciences of Food Production, Italian National Research Council, Via Celoria 2, Milan 20133, Italy; E-Mails: stefano.morandi@ispa.cnr.it (S.M.); tiziana.silvetti@ispa.cnr.it (T.S.); 2 Department of Food, Environmental and Nutritional Sciences, State University of Milan, Via Celoria 2, Milan 20133, Italy; E-Mails: veronica.rosi@unimi.it (V.R.); stefano.cattaneo@unimi.it (S.C.); luisa.pellegrino@unimi.it (L.P.)

**Keywords:** lysozyme, turbidimetric assay, hen egg white protein, HPLC, SDS-PAGE

## Abstract

Hen egg-white lysozyme (LSZ) is currently used in the food industry to limit the proliferation of lactic acid bacteria spoilage in the production of wine and beer, and to inhibit butyric acid fermentation in hard and extra hard cheeses (late blowing) caused by the outgrowth of clostridial spores. The aim of this work was to evaluate how the enzyme activity in commercial preparations correlates to the enzyme concentration and can be affected by the presence of process-related impurities. Different analytical approaches, including turbidimetric assay, SDS-PAGE and HPLC were used to analyse 17 commercial preparations of LSZ marketed in different countries. The HPLC method adopted by ISO allowed the true LSZ concentration to be determined with accuracy. The turbidimetric assay was the most suitable method to evaluate LSZ activity, whereas SDS-PAGE allowed the presence of other egg proteins, which are potential allergens, to be detected. The analytical results showed that the purity of commercially available enzyme preparations can vary significantly, and evidenced the effectiveness of combining different analytical approaches in this type of control.

## 1. Introduction

Lysozyme (LSZ, muramidase, EC 3.2.1.17) is one of the most extensively studied antimicrobial enzymes. It occurs in several mammalian secretions (milk, saliva, tears) and also in hen egg white (HEW), which represents the raw material of choice for the production of LSZ on an industrial scale [1]. This low molecular weight enzyme (14,307 Dalton) consists of 129 amino acids cross-linked by four disulphide bridges, and shows lytic activity on the β(1→4) glycosidic bond between N-acetyl-D-glucosamine and N-acetylmuramic acid in the cell wall of bacterial species, particularly Gram-positive microorganisms such as lactic acid bacteria (LAB) and Clostridia. The main use of LSZ in the food industry is related to limiting the proliferation of LAB spoilage in the production of wine and beer [2,3], and to inhibiting Clostridia growth during cheese maturation [4]. Butyric acid fermentation in cheese (late blowing), caused by the outgrowth of clostridial spores present in raw milk, most commonly originating from silage, can cause considerable product loss, especially in the production of hard and extra-hard cheeses. The most common strategies adopted to prevent late blowing defects are bactofugation and microfiltration of milk, and the addition of nitrates or LSZ [4,5,6]. Lysozyme has been shown to be particularly effective in cheeses like Edam, Gouda, Cheddar, Emmental, Asiago, Grana Padano, Montasio, Provolone, Manchego, Brebiou and Castelões [6,7,8]. France, in 1981, was the first country to allow the industrial application of LSZ in cheese production [6], and nowadays LSZ is permitted as a preservative (E1105) in ripened cheeses, in accordance with current EU legislation [9] and the Codex Alimentarius [10]. The estimated content of LSZ in cheese roughly ranges between 100 and 350 mg per kilogram [7,11]. 

Egg products count among the most common causes of food allergies, with the estimated prevalence of egg allergies being 2%–3% in children and in adults [12]. For this reason, the use of LSZ must be declared to comply with EU allergen labelling instructions [13]. 

Previous studies have reported the antigenicity and allergenicity of egg white proteins, suggesting that ovotransferrin and ovomucoid have important implications in the anaphylactic reaction to eggs while ovoalbumin and ovomucoid are crucial for atopic reactions [14]. At present, the role of LSZ itself as an allergen is still controversial. In fact, some studies have come to the conclusion that LSZ is only a weak allergen [15,16], while others support the opposite conclusion [17,18]. The Joint FAO/WHO Expert Committee on Food Additives [19] advises carrying out a turbidimetric analysis to determine LSZ potency. The method is based on LSZ lytic activity on the bacterial cells of *Micrococcus luteus* (*Micrococcus lysodeikticus*) quantified by turbidimetric analysis [20,21]. Indeed several methods have been developed to identify and quantify the LSZ molecule, including Sodium Dodecyl Sulphate-Polyacrylamide Gel Electrophoresis (SDS-PAGE) [22], High Performance Liquid Chromatography with fluorescence detection (HPLC-FLD) [11], Enzyme-Linked Immunosorbent Assay (ELISA) [23], immunocapture mass spectrometry [24], and surface-enhanced mass spectrometry [7,25]. The HPLC-FLD method proposed by Pellegrino and Tirelli [11] to quantify LSZ in milk and dairy products was recently published as an ISO Technical Specification [26]. 

The EU Commission Regulation No 231/2012 [27] recently defined specifications for food additives, including LSZ. The aim of this work was to assess the suitability of different analytical approaches to evaluate the purity and molecular integrity of the enzyme, with respect to its activity as determined by the turbidimetric assay. 

## 2. Results and Discussion

### 2.1. Purity of Commercial Lysozyme Preparations

The gross composition of 17 LSZ preparations marketed in different countries, and intended for use in the dairy industry (Table 1), was evaluated with respect to the most relevant specifications provided in Commission Regulation 213/2012 [27]. 

**Table 1:** Origin, type, and composition characteristics of the commercial lysozyme preparations. Values not complying with specifications of EU Reg. 231/2012 are in bold.

Sample	Country of Purchase ^1^	Type of preparation	Water (g/100 g)	Nitrogen (g/100 g)	Total Protein ^2^ (g/100 g)	Sugars ^3^ (g/100 g)	HEW proteinsother than LSZ ^4^ (+/−)
1	Netherland (M)	Granular	5.00	17.38	91.9	n.d.	+
2	Italy (D)	Granular	**6.71**	16.73	88.5	n.d.	+
3	Italy (D)	Granular	**6.67**	16.75	88.6	n.d.	-
4	Germany (M)	Powder	**7.61**	**16.48**	87.2	0	+++
5	Italy (D)	Granular	**6.37**	16.81	88.9	n.d.	+
6	Italy (D)	Powder	5.07	**15.67**	82.9	7.6 (g)	+++
7	Italy (M)	Granular	**8.66**	16.86	89.2	n.d.	+
8	Italy (D)	Granular	5.10	16.77	88.7	n.d.	++
9	Switzerland (M)	Granular	5.70	17.20	91.0	n.d.	-
10	Italy (D)	Granular	5.00	17.44	92.3	n.d.	++
11	Germany (D)	Powder	**8.06**	16.84	89.1	n.d.	+
12	Spain (D)	Granular	**6.60**	**12.53**	66.3	24.0 (g)	-
13	Spain (D)	Liquid ^5^	n.d.	4.15	22.0	n.d.	+
14	Spain (D)	Granular	5.50	**13.31**	70.4	17.3 (s)	++
15	Belgium (M)	Powder	4.10	17.14	90.7	n.d.	+
16	Canada (M)	Granular	5.40	17.31	91.6	n.d.	+
17	Poland (M)	Powder	5.00	**16.11**	85.2	0	++++

^1^ (M): manufacturer or (D): distributor; ^2^ By calculation (N × 5.29); ^3^ (g): glucose; (s): starch and trace levels of maltose, maltotriose and glucose; ^4^ As visually evaluated from SDS-PAGE gels; n.d.: not determined; ^5^ Values are expressed as g/100 mL.

With the exception of the liquid sample, the water content varied from 4.1 to 8.7 g/100 g (Table 2). Despite the wide variability, many samples did not comply with the maximum limit of 6%. It should be mentioned here that the water content was measured by gravimetric oven drying, whereas the legal limit refers to Karl Fischer titration. This latter method is much more accurate for products like dried milk and lactose powder [28] as it also determines the water of crystallization, however this is not present in our samples. Moisture has a relevant effect on the stability of solid-state enzymes during storage, and an increase in LSZ aggregation with increasing humidity was evidenced by Separovich, Lam, Ke, and Chan [29]. On the other hand, the dehydration of LSZ caused a loss of activity, apparently due to the removal of water molecules residing functionally in the active site [30]. These aspects will be further discussed throughout this paper.

**Table 2:** Potency (microbiological activity) and concentration (HPLC) of lysozyme in the commercial preparations.

Sample	Type of preparation		Anhydrous basis (mg/g)	Product basis ^1^ (g/100 g product)		Protein basis (g/100 g protein)	
			Potency	Potency	Concentration	Potency	Concentration
1	Granular		1008	96 ± 1.73	100 ± 0.71	104	109
2	Granular		929	87 ± 0.06	83 ± 0.70	98	94
3	Granular		1040	97 ± 1.58	97 ± 2.11	110	109
4	Powder		872	81 ± 0.13	75 ± 0.70	92	86
5	Granular		968	91 ± 0.46	86 ± 1.68	102	96
6	Powder		753	71 ± 0.51	66 ± 1.95	86	80
7	Granular		949	87 ± 0.17	80 ± 1.36	97	90
8	Granular		985	93 ± 0.23	95 ± 4.28	105	107
9	Granular		1027	97 ± 0.02	99 ± 2.10	106	109
10	Granular		1001	95 ± 0.41	95 ± 1.42	103	103
11	Powder		960	88 ± 0.32	79 ± 2.13	99	89
12	Granular		747	70 ± 2.90	76 ± 0.69	105	114
13 ^2^	Liquid		n.d.	26 ± 0.56	24 ± 1.41 ^3^	118	108 ^3^
14	Granular		746	70 ± 1.13	70 ± 0.70	100	99
15	Powder		985	94 ± 0.07	84 ± 0.71	104	93
16	Granular		1025	97 ± 1.40	93 ± 2.11	106	101
17	Powder		643	61 ± 0.24	52 ± 0.72	72	61

^1^ Mean values ± relative standard deviation; ^2^ Values on product basis are expressed as g/100 mL; ^3^ Area of the two main peaks was considered (see paragraph 2.2); n.d.: not determined.

The EU product specification for LSZ provides that the nitrogen content will be in the range 16.8%–17.8%. Five samples out of 17 proved to be below the range, none were above (Table 1). Although there is no reference to protein content in the LSZ specification, this was calculated in order to have a better evaluation of product purity. We derived the specific nitrogen-to-protein conversion factor of 5.29 from the atomic composition of LSZ [31], instead of using the general value of 6.25 commonly adopted for food protein, as this last led to large overestimation. 

The protein content varied from 88.5 to 92.3 g/100 g in samples with a nitrogen content within the legal limits, irrespective of whether the product was powdered or granular. In the liquid sample (n.13) the protein content was 22 g/100 mL, corresponding to the content declared by the manufacturer on the technical sheet, whereas in the five remaining samples collected in Spain (samples 12 and 14), Germany (n.4), Italy (n.6) and Poland (n.17), it ranged from 66.3% to 87.2% (Table 1). Based on such low levels, and taking the respective water content into consideration, it was hypothesized that other ingredients had been added to the commercial LSZ preparation as excipients. Therefore, these five samples were analyzed for the presence of sugars using an HPLC method that allows both mono- and polysaccharides to be quantified. The presence of 7.6% and 24% glucose was detected in samples 6 and 12, respectively, while starch (17%), maltotriose, and traces of glucose and maltose were found in sample 14 (Table 1). These saccharides are not among those commonly used as stabilizers in protein preparations, hence their presence could have been due to an intentional addition as low cost fillers. As stated by Wang [32], disaccharides such as sucrose are the most effective in stabilizing protein during dehydration steps, as large saccharides are ineffective and glucose is extremely reactive with free amino groups of lysine and arginine to form adducts *via* the Maillard reaction. Surprisingly, no sugars were found in sample 17, despite its very low protein content. This sample was analyzed for the presence of other common excipients such as free amino acids, calcium chloride, sodium chloride and potassium chloride, and all these substances were found at the expected trace levels (not shown).

**Figure 1:**
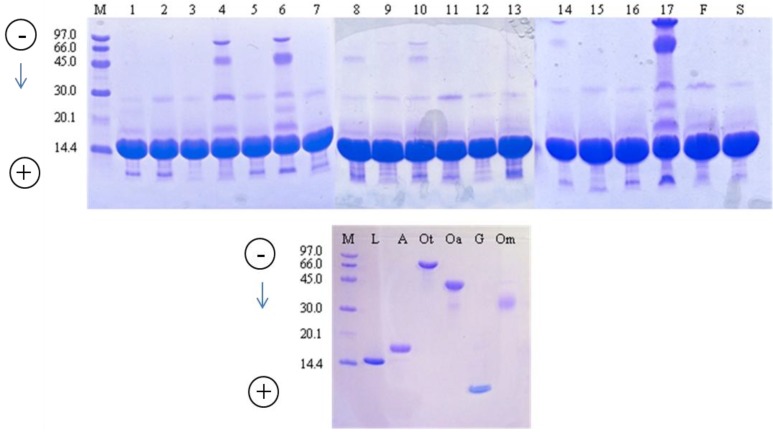
Sodium dodecyl sulphate (SDS) gel electrophoresis of commercial lysozyme preparations. Lane M: molecular weight markers; Lane from 1 to 17: lysozyme samples; F: FIP lysozyme standard; S: Sigma-Aldrich lysozyme; L: lysozyme; A: avidin; Ot: ovotransferrin; Oa: ovalbumin; G: gallin; Om: ovomucoid.

SDS-PAGE was used to evaluate the protein pattern of the commercial LSZ preparations (Figure 1). Electrophoretic bands were identified by running the pure HEW proteins separately. Although the LSZ band (approx. 14 kDa) markedly dominated in all of the samples, the presence of other HEW proteins was also evident. Samples 3, 9 and 12 proved to be the most pure, only weak bands of LSZ dimer and avidin being detected (Table 2) along with that of LSZ. Bands of LSZ dimer, avidin and gallin were detected in the remaining commercial samples, and additional bands were present in seven of them. Ovalbumin and/or ovotransferrin was present in samples 4, 6, 8, 10, 14 and 17. Sample 17 was by far the worst commercial LSZ preparation, being heavily contaminated by several HEW proteins other than LSZ.

SDS-PAGE proved to be a useful technique to detect the presence of undesired HEW proteins in commercial preparations, and this aspect could be of concern as LSZ is used in several food products. As already mentioned, the band corresponding to MW of 28,600 Da was assumed to be the LSZ dimer. In fact, the formation of intermolecular covalent bonds was reported to occur in LSZ submitted to dry-heating [33], and it was recently proposed that the chemical pathway involves the formation of a succinimide ring [34]. Interestingly, a weak band corresponding to the LSZ dimer was detected also in the pure LSZ standard (lane S, Figure 1), confirming that even mild technological treatments or prolonged storage at a low water-activity level may induce the reaction [34]. Several researchers have shown the lytic activity of LSZ dimer to be the same as that of the monomer [33,35]. Therefore, the presence of the dimerized form should not result in a lower potency of the preparations. 

Avidin with an estimated molecular mass of 16 kDa was detected in all the samples, although its theoretical molecular mass is 68.3 kDa [36]. Korpela [37] demonstrated that, under reducing conditions, avidin is fragmented into four monomers of MW from 15.6 to 15.9 kDa. Avidin has antimicrobial activity due to its ability to bind to various gram-negative and gram-positive bacteria, including *Escherichia coli* K-12, *Klebsiella pneumoniae*, *Serratia marcescens*, *Pseudomonas aeruginosa*, *Staphylococcus aureus* and *S. epidermidis* [37]. 

No data are available on the allergenic nature of avidin. In contrast, ovotransferrin (70 kDa) and ovalbumin (45 kDa) are considered the major allergens in the egg white fraction [14]. These proteins were observed in preparations 5 and 6 (Figure 1). In three samples (samples 1, 6, 17) a band of approximately 23 kDa, which could be attributed to the Ch21 protein [38], was detected. The Ch21 protein, which belongs to the lipocalin family, is located in albumen and has been reported as allergenic [39]. In all the LSZ preparations, except for samples 3 and 9, the egg protein named gallin (7 kDa) was observed (Figure 1). Gallin was first identified in the analysis of HEW using a proteomic approach [40]. It was named gallin because of its homology to meleagrin, a peptide previously discovered as a contaminant in turkey ovomucin preparation, and to cygnin, discovered in the preparation of black swan LSZ [41,42]. The function of this peptide has not yet been identified, but it has a potent antimicrobial activity, particularly against *Escherichia coli* [43]. Proteins such as ovomucin and ovostatin, of molecular mass higher than 100 kDa, cannot be observed using this type of polyacrylamide gel because it only permits the resolution of proteins smaller than 100 kDa [44]. The data showed that, at the industrial level, the LSZ purification process is sometimes disregarded by manufacturers, due to the high costs and significant amounts of potentially allergenic proteins that can be retained in the commercial preparation. The presence of residual egg proteins other than LSZ can be detected with good sensitivity by SDS-PAGE, although the quantitative evaluation is far from being accurate.

### 2.2. Lysozyme Concentration and Potency

The specifications laid down by EU Regulation 231/2012 provide microbiological assay as the only parameter directly related to LSZ content in the finished product, and the minimum limit is 950 mg/g on the anhydrous basis. 

Considering the microbiological activity determined by the turbidimetric assay [19], the derived potency values ranged between 643 and 1,040 mg/g on the anhydrous basis (Table 2). Six preparations did not comply with the legal minimum content and, as expected, the lowest values of potency were found in samples with low nitrogen content, confirming the presence of substances other than LSZ. 

LSZ concentration can be directly determined in milk and cheese by a dedicated HPLC-FLD method widely adopted by the dairy industry and official control organizations [26]. We determined the LSZ concentration in all the commercial preparations by using this method and a pure LSZ standard for quantification. Chromatographic separation was originally optimized to quantify added LSZ in ripened cheeses [11], hence without the interference of other milk proteins or peptides. Under these conditions, HEW proteins other than LSZ are eluted elsewhere in the chromatogram (not shown) and were not detected. The LSZ concentration was in the range of 79 to 100 g/100 g product in those formulations complying with the limits provided by the EU Regulation for nitrogen content, whereas lower levels were found in the irregular samples (Table 1 and Table 2). 

**Figure 2:**
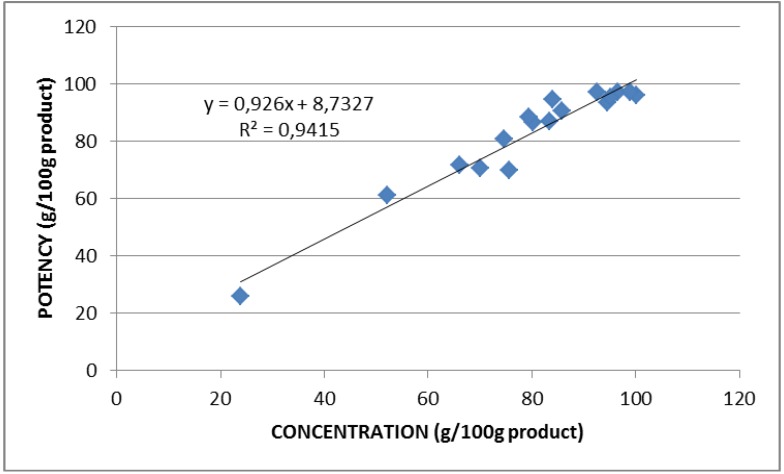
Correlation between potency (microbiological activity) and concentration (HPLC) of lysozyme in commercial preparations.

Overall, the concentration values were in very good agreement with the potency values, despite they were obtained using completely different approaches. In fact, the former implies the evaluation of the LSZ molecule in the preparation, the latter its enzymatic activity. The high correlation between the two determinations is shown in Figure 2. On the anhydrous basis the potency data highlighted those samples (2, 4, 6, 12, 14, 17) not meeting the required EU Regulation specification, while figures expressed on product basis provide the real LSZ content in the commercial preparation. On the other hand, low values expressed on protein basis demonstrate the presence of proteins other than LSZ (samples 4, 6, 17). Values expressed on protein basis nearest 100 in combination with low values of potency on an anhydrous basis indicate the presence of substances other than protein, as evidenced in samples 6, 12, 14.

**Figure 3:**
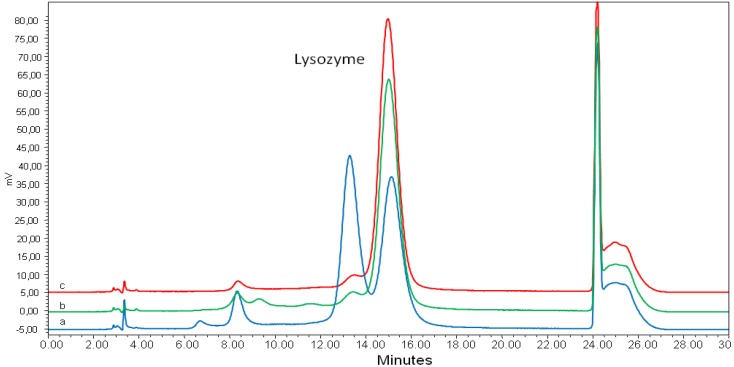
HPLC patterns of lysozyme in commercial preparations nr 13 (pattern **a**) and nr 15 (pattern **b**) and in lysozyme standard (pattern **c**).

The LSZ concentration in the liquid formulation (sample 13) was 12 g/100 mL. However, this sample presented an incredibly unusual HPLC pattern, with a large peak eluting at 13 min just before the LSZ peak (Figure 3, pattern a). This peak is usually very small in the commercial preparations (pattern b, referring to sample 15), pure standard (pattern c), as well as in milk and cheese samples [11]. Thus it is disregarded in LSZ quantification in accordance with the ISO-IDF method [26], despite the same UV spectrum as LSZ. By considering both the peaks of sample 13 in the calculation, we found a LSZ concentration of 24 g/100 mL, which is very close to both the expected value according to product specifications (*i.e.*, 22 g/100 mL) and potency value (Table 2). Interestingly, we found a large amount of the peak eluting before LSZ in a very old (10 years) powder preparation, the LSZ peak decreasing proportionally. The identity of this peak is under investigation by HPLC/ESI MS. Preliminary results showed the presence of LSZ with both *m/z* of 14290 and 14292, whereas the main peak showed the expected *m/z* 14306 (data not shown). Schneider *et al.* [25], suggested a conformational isomer of LSZ to elute in this peak which progressively increased in a LSZ solution heated at 99 °C for up to 120 min, as well as in cheese samples during storage. On the other hand, Desfougères *et al.* [34] studied the effect of heating LSZ in the dry state (80 °C for up to 7 days) under mildly acidic conditions. Using cation exchange liquid chromatography and MS, these authors demonstrated that succinimide derivatives formed in LSZ from ASP and ASN residues without affecting the secondary and tertiary structure of the molecule but decreasing its lytic activity against *Micrococcus lysodeikticus*. 

## 3. Experimental

### 3.1. Lysozyme Samples and Reference Materials

A total of 17 different LSZ preparations, collected from 8 different countries, were purchased from either the producer or the commercial distributor (Table 1). A 10-year old LSZ preparation was provided by the manufacturer. Pure HEW LSZ L-6876 from Sigma Aldrich (St. Louis, MO, USA) and from FIP (International Commission on Pharmaceutical Enzymes, Centre for Standards, Harelbekestraat, Belgium) were taken as working standards for HPLC-FLD and potency determination respectively. Pure avidin, ovalbumin, ovomucoid, and ovotransferrin, were purchased from Sigma. Gallin was kindly supplied by the QA/QC laboratory of Neova Technologies, Abbotsford, BC, Canada.

### 3.2. Composition Analysis

Water content was determined gravimetrically, with the exclusion of the liquid sample. A glass dish and its lid were dried overnight in a drying oven at 102 ± 2 °C. The glass dish was allowed to cool to room temperature in a desiccator. Five grams of LSZ preparation were weighed into the glass dish to the nearest 0.1 mg. The loaded dish with open lid was dried in the oven at 102 ± 2 °C for 3 h (constant weight), then it was closed, cooled in the desiccator and weighed. The total nitrogen content was determined by the Kjeldahl method [45] and 5.29 was used as the conversion factor to protein. Sugar content was determined by HPLC, with refractive index detection on selected samples according to the Standard ISO 22662: IDF 198 – 2007 [46]. About 250 mg of LSZ preparation were weighed into a 100-mL one-mark volumetric flask, added with 80 mL of water and 5 mL Biggs-Szijarto solution [46] to precipitate out protein, and diluted to the 100-mL mark with water. The sample was kept at room temperature for 30 min, then was centrifuged at 5,000 *g* for 10 min, filtered on PVDF filter and analyzed by HPLC. Mean values of two replicates were considered. Repeatability of the data was within the limits provided by the respective reference methods.

### 3.3. SDS-PAGE Analysis

SDS-PAGE was performed on a PhastSystem electrophoresis apparatus (Amersham Biosciences, Buckinghamshire, UK) using the commercial Homogeneous 20% polyacrylamide precast gels and PhastGel SDS buffer strips (Amersham Biosciences). Electrophoretic and staining conditions with PhastGel Blue R were those recommended by the manufacturer. Two-hundred µL of LSZ solution (20 mg/mL) was dissolved in 200 µL of 10 mM tris(hydroxymethyl) aminomethane–HCl sample buffer (pH 8.0), containing 2.5% SDS (Merck, Darmstadt, Germany), 10 mM EDTA (Merck) and 5% 2-mercaptoethanol (Merck), and was heated at 99 °C for 10 min. A 1-µL aliquot of each sample was loaded on the gel. Molecular weight standard proteins (GE Healthcare, Little Chalfont, UK) were α-lactalbumin (14.4 kDa), soy trypsin inhibitor (20.1 kDa), carbonic anhydrase (30.0 kDa), ovalbumin (45.0 kDa), bovine serum albumin (66.0 kDa) and phosphorylase b (97.0 kDa). Identification of other egg proteins in the samples was achieved by running pure avidin, gallin, ovomucoid, and ovotransferrin under the same conditions.

### 3.4. Determination of Lysozyme Potency

The microbiological activity (potency) of LSZ preparations was determined by the turbidimetric assay, according to JECFA method [19]. The cell suspension of *Micrococcus luteus* (*M. lysodeikticus*) ATCC 4698 (80 mg in 200 mL) was obtained by rehydration of lyophilized commercial preparation (Sigma) in M/15 phosphate buffer (pH 6.6 ± 0.1). A calibration curve was prepared with the FIP enzyme of known activity. Required amounts of LSZ were added in the test-tubes in order to obtain final quantities of 0.20, 0.28 and 0.40 μg/mL. At exactly 30 s intervals, 5 mL of the suspension of *M. luteus* were added to each test-tube, and incubated in a water bath at 37 ± 0.5 °C for exactly 12 min. After incubation, the test-tubes were removed from the water bath in the same order as they were put in and the absorbance at 450 nm was recorded against the buffer solution every 30 s, using a UVIDEC320 spectrophotometer (Jasco, Easton, PA, USA). The potency was estimated as the mean value of three replicates for each LSZ sample.

### 3.5. HPLC-FLD Analysis

Commercial preparations of LSZ were analyzed by HPLC-FLD following the procedure described in the ISO method [26]. Ten milligrams (or 10 µL for liquid preparation) of LSZ preparation were dissolved in 10 mL of water and, after complete solubilisation, 80 µL of the solution were further diluted to 10 mL with 1 mol/L sodium chloride. The chromatographic separation was performed with an Alliance system (Waters, Milford, MA, USA) equipped with a L-2485 fluorescence detector (VWR, Milan, Italy) operating at 280 nm excitation, and 340 nm emission and a 2996 DAD (Waters). The LSZ concentration was calculated by single point calibration using the pure LSZ from Sigma, as provided by the reference method, and results are mean values of duplicated analyses.

## 4. Conclusions

The analytical results of this study evidenced that the commercially available preparations vary significantly in terms of purity, LSZ concentration and potency. This work has demonstrated that the HPLC method adopted by ISO allows the LSZ concentration to be determined with accuracy and data are significantly correlated to those obtained by the turbidimetric assay advised by FAO/WHO. Furthermore, the HPLC method gives evidence of molecular modifications that can occur during the production process and conservation. SDS-PAGE is suitable for detecting the presence of undesired egg proteins. The rather widespread presence of residual HEW proteins other than LSZ suggests that the control of this aspect should be performed regularly when LSZ is intended as a food additive. To this end, reliable and sensitive analytical methods are indispensable tools. Finally, some of the samples analyzed did not comply with the specifications of EU Regulation 231/2012, most frequently because nitrogen content and enzyme potency were below their respective minimum limits. In some cases this was due to the undeclared presence of saccharides, which proved to replace up to 25% of the enzyme in the product. 
